# SWIR Photodetection and Visualization Realized by Incorporating an Organic SWIR Sensitive Bulk Heterojunction

**DOI:** 10.1002/advs.202000444

**Published:** 2020-05-29

**Authors:** Ning Li, Zhaojue Lan, Ying Suet Lau, Jiajun Xie, Dahui Zhao, Furong Zhu

**Affiliations:** ^1^ Department of Physics Research Centre of Excellence for Organic Electronics Institute of Advanced Materials, and State Key Laboratory of Environmental and Biological Analysis Hong Kong Baptist University NT Hong Kong China; ^2^ Beijing National Laboratory for Molecular Sciences Centre for Soft Matter Science and Engineering Key Laboratory of Polymer Chemistry and Physics of the Ministry of Education College of Chemistry Peking University Beijing 100871 China

**Keywords:** organic photodetectors, solution fabrication, SWIR photodetection, SWIR visualization

## Abstract

Short‐wavelength infrared (SWIR) photodetection and visualization has profound impacts on different applications. In this work, a large‐area organic SWIR photodetector (PD) that is sensitive to SWIR light over a wavelength range from 1000 to 1600 nm and a SWIR visualization device that is capable of upconverting SWIR to visible light are developed. The organic SWIR PD, comprising an organic SWIR sensitive blend of a near‐infrared polymer and a nonfullerene n‐type small molecule SWIR dye, demonstrates an excellent capability for real‐time heart rate monitoring, offering an attractive opportunity for portable and wearable healthcare gadgets. The SWIR‐to‐visible upconversion device is also demonstrated by monolithic integration of an organic SWIR PD and a perovskite light‐emitting diode, converting SWIR (1050 nm) to visible light (516 nm). The most important attribute of the SWIR visualizing device is its solution fabrication capability for large‐area SWIR detection and visualization at a low cost. The results are very encouraging, revealing the exciting large‐area SWIR photodetection and visualization for a plethora of applications in environmental pollution, surveillance, bioimaging, medical, automotive, food, and wellness monitoring.

## Graphical abstract

A high‐performance organic shortwave infrared (SWIR) photodetector (PD) is developed for photodetection and visualization. The organic SWIR PD is sensitive over the wavelength range from 1000 to 1600 nm, having a specific detectivity of >10^9^ Jones. SWIR‐to‐visible upconversion demonstrated in this work has advanced the capability of detecting the longest infrared wavelengths using an organic PD reported in the field.

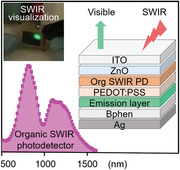

## 1 Introduction

Short‐wave infrared (SWIR) photodetectors (PDs) are able to extend the human vision by converting invisible SWIR to visible light for different applications. The human vision is quite limited and within a narrow visible light wavelength range from 350 to 700 nm. The detection and visualization of the electromagnetic waves with wavelengths beyond the human vision, e.g., visualizing near‐infrared (NIR) or SWIR light, are critical and challenging. The SWIR over the wavelength range from 1000 to 3000 nm covers the second and third biological window.^[^ ^1^ ^]^ SWIR photodetection and visualization in this wavelength range plays a critical role for a plethora of applications in environmental pollution, surveillance, bioimage, medical, agricultural, automotive, food, and wellness monitoring, due to its unique characteristics, e.g., reduced light scattering and absorption,^[^ ^2^ ^]^ deep penetration depth in biotissues,^[^ ^3^, ^4^, ^5^ ^]^ and reduced phototoxicity.^[^ ^6^ ^]^ The commercially available III–V compound semiconductor‐based SWIR PDs are small in size, rigid, and costly, having limitation for use in flexible and wearable devices. Very recently, a novel high‐performance bias‐switchable spectral response organic PD in two distinct NIR and visible light bands has been developed, potentially attractive for applications in wellness and security monitoring.^[^ ^7^ ^]^ However, it is still an open challenge for preparing a high‐performance SWIR PD and visualizing device over a large area at a low cost. The recent advances in low bandgap organic semiconductors and solution fabrication technologies have provided an encouraging pathway for attaining solution‐processable flexible and large‐area SWIR detection devices at a low cost. The huge materials varieties and tunable electronic/optical properties of the organic semiconductors^[^ ^8^ ^]^ with desired low bandgap and high absorption coefficient offer an attractive option for use in high‐performance organic SWIR photodetection and visualization.

Visualizing NIR light has been demonstrated using the ocular injectable photoreceptor‐binding upconversion nanoparticles.^[^ ^9^ ^]^ Although the anchored nanoparticles on retinal photoreceptors for visualizing NIR light create negligible side effects, there are still potential risks. The visualizing NIR or SWIR light through noninvasive upconversion approach would be more attractive for practical applications, converting the low energy infrared photons to high energy visible photons that can be received by the retina and perceived by our brain. The conventional infrared visualization systems use an inorganic semiconductor, e.g., InGaAs, PD array,^[^ ^10^ ^]^ involving a high‐cost fabrication and complicated data process. The operation of the inorganic semiconductor SWIR PDs requires cooling^[^ ^11^ ^]^ for reducing the thermal noise. Nanoparticles incorporating rare earth elements for upconversion process have been reported for applications in hyperspectral remote sensing, bioimaging, and surveillance. The operation of these nanoparticle‐based PDs requires a high intensity of infrared source, the toxicity of the rare earth containing nanoparticles in biotissues^[^ ^2^, ^5^ ^]^ is another critical issue limiting the applications in agricultural, food, and wellness monitoring. An infrared to visible light upconversion device, comprising a monolithic integration of an infrared PD and an organic light‐emitting diode (OLED), is gradually revealing its potential for use in infrared photodetection and visualization. In an upconversion device, the photocurrent generated in the infrared PD controls the charge injection in the LED in the presence of the invisible light. The visible light emission in LED can be observed in area where the effective charge injection from infrared PD takes place, such that the objects reflecting or illuminating invisible infrared light can be visualized. However, realization of SWIR photodetection and visualization by incorporating an organic SWIR sensitive bulk heterojunction (BHJ) is less reported, due to the challenge in processing low bandgap organic semiconductors with suitable energy levels.

In this work, we report our effort to develop a solution‐processable large‐area organic SWIR PD that is sensitive to the invisible light over a wavelength range from 1000 to 1600 nm and a SWIR visualizing device that is capable of upconverting SWIR (1050 nm) to visible light (516 nm). An organic SWIR sensitive BHJ, prepared using a blend of a diketopyrrolopyrrole‐dithienylthieno[3,2‐b]thiophene (DPP‐DTT) polymer donor and a hetero‐polycyclic aromatic compound SWIR dye, was used for application in SWIR photodetection and visualization. It shows that the PDs made with the organic SWIR sensitive BHJ are a suitable choice for a portable photoplethysmography (PPG) sensor, enabling the real‐time heart rate monitoring. An organic SWIR visualizing device has been demonstrated by monolithic integration of an organic sensitive SWIR BHJ and a cesium lead bromide (CsPbBr_3_) perovskite LED. The organic BHJ in the SWIR‐to‐visible upconversion device also acts as an optical coupling layer to enhance the visible light emission from the perovskite LED.

## 2 Results and Discussion

### 2.1 SWIR Photodetection

**Figure 1:**
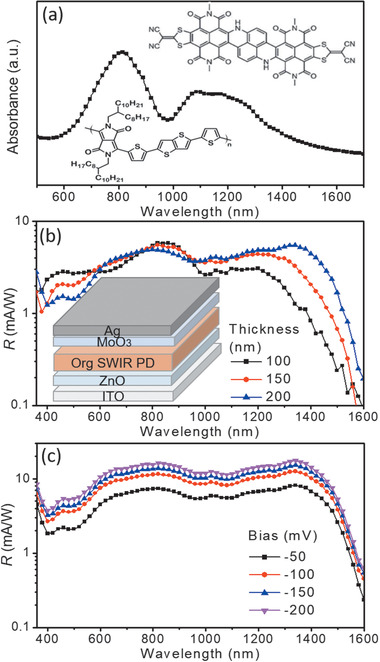
Photoresponses of the organic SWIR PDs. a) The absorption spectrum measured for the DPP‐DTT:SWIR dye blend layer deposited on glass. The insets in (a): molecule structures of the DPP‐DTT polymer and the SWIR dye. b) *R*(*λ*) of the organic SWIR PDs, with different BHJ layer thicknesses, as a function of the wavelength. The inset in (b): a schematic cross‐sectional view of an organic SWIR PD. c) *R*(*λ*) measured for an organic SWIR PD with a 200 nm thick BHJ layer operated under different reverse biases.

The organic SWIR sensitive BHJ, comprising a DPP‐DTT polymer donor and an n‐type hetero‐polycyclic aromatic compound SWIR dye, has a pronounced absorption extended to the SWIR region. The absorption spectrum measured for the DPP‐DTT:SWIR dye blend layer is plotted in **Figure** **1**a, revealing its remarkable absorption over the long wavelength range from 600 to 1600 nm, with two prominent absorption peaks located at 820 and 1100 nm, respectively. The molecule structures of the polymer donor and SWIR dye are shown in the inset in Figure 1a. The DPP‐DTT polymer donor has a peak absorption at 820 nm with an absorption edge extended to 950 nm.^[^ ^12^, ^13^ ^]^ The SWIR sensitive BHJ layer has a pronounced absorption over the long wavelength range from 1000 to 1600 nm, contributed mainly by the SWIR dye due to its low bandgap.^[^ ^14^ ^]^ The photoresponsivity, *R*(*λ*), of the organic SWIR PDs, comprising a layer configuration of ITO/ZnO/DPP‐DTT:SWIR dye/MoO_3_/Ag, was analyzed. *R*(*λ*) of a PD is related to its external quantum efficiency (EQE), in the form of *R*(*λ*) = EQE/(*hc*/*λ*), where *h* is Planck's constant, *c* is the speed of light in vacuum, and *λ* is the wavelength of the incident light. The unit for *hc*/*λ* is eV. *R*(*λ*) measured for the organic SWIR PDs with different BHJ thicknesses of 100, 150, and 200 nm, annealed at 150 °C, is plotted in Figure 1b. A schematic cross‐sectional view of an organic SWIR PD is shown in the inset in Figure 1b. *R*(*λ*) of the organic SWIR PDs over the wavelength range from 380 to 1600 nm was characterized. The photosensitivity of the organic SWIR PDs increases with the BHJ layer thickness, particularly over the long wavelength range from 1000 to 1600 nm, due to the enhanced absorption in the thick BHJ active layer. The effect of the postannealing of the BHJ on *R*(*λ*) of the organic SWIR PDs made with a 200 nm thick BHJ was analyzed. An optimal annealing temperature of 150 °C was used in the device fabrication, the results are shown in Figure S1 (Supporting Information).

*R*(*λ*) measured for the organic SWIR PD with a 200 nm thick BHJ under different reverse biases of −50, −100, −150, and −200 mV is shown in Figure 1c. A wavelength independent broadband enhancement in *R*(*λ*), increasing with the reverse bias, was observed, revealing an efficient photogenerated charge transportation and charge extraction in the organic SWIR PD operated under a higher reverse bias. Organic photodetectors (OPDs) developed in this work exhibit a broadband photoresponse over the wavelength range from 400 to 1600 nm. *R*(*λ*) of the organic SWIR PDs, measured under different intensities of the light sources with different peak emission wavelengths of 365, 450, 850, and 1050 nm, as a function of reverse bias is plotted in Figure S2 (Supporting Information). A relatively higher *R*(*λ*) was observed for the organic SWIR PDs operated under a high reverse bias. The results reveal that the *R*(*λ*) of the organic SWIR increased with decrease in the intensity of the SWIR source. This is because the photoresponsivity of a PD is inversely proportional to the intensity of the incident light, e.g., *R*∝*P* ^−*α*^, where *P* is the intensity of the incident light. The exponent *α* is usually smaller than unity, resulting in the *R*(*λ*) increasing with decrease in the intensity of the incident light.

**Figure 2:**
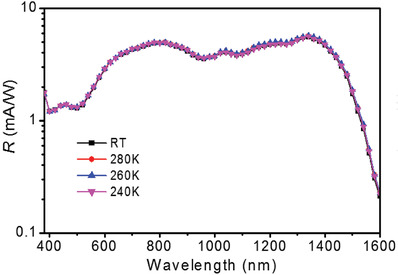
Temperature independent *R*(*λ*) of the organic SWIR PD. *R*(*λ*) measured for an organic SWIR PD, with a 200 nm thick BHJ, operated at different temperatures.

The effect of the operation temperature on the performance of the organic SWIR PDs was analyzed by monitoring the *R*(*λ*) measured for the PDs operated at different temperatures. It shows that *R*(*λ*) of an organic SWIR PD with a 200 nm thick BHJ was almost identical over an operation temperature range from 240 K to room temperature, as shown in **Figure** **2**. The cooling of the conventional SWIR PDs, made with the inorganic III–V compound semiconductors, is required to reduce the thermal noise. It shows that the photoresponse of the DPP‐DTT:SWIR dye‐based organic SWIR PDs has a temperature insensitive feature, which is very useful for practical applications. The organic SWIR PDs with an almost temperature independent *R*(*λ*) are closely associated with the suppression of thermal‐assisted charge transport in the BHJ.

**Figure 3:**
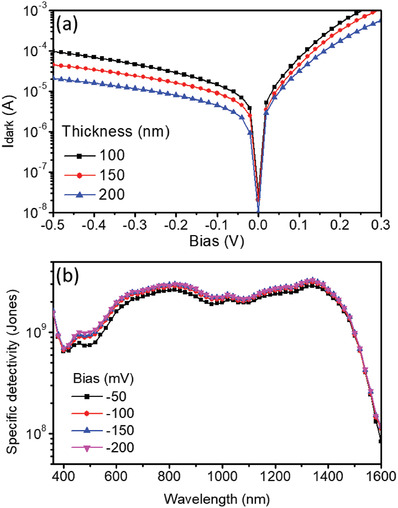
Dark current and D* analyses. a) *I*_dark_−*V* characteristics measured for the organic SWIR PDs with different BHJ layer thicknesses of 100, 150, and 200 nm. b) D* calculated for an organic SWIR PD, made with a 200 nm thick BHJ, operated under different reverse biases.

The dark current, dominating the noise level of the OPDs,^[^ ^7^ ^]^ is considered as shot noise when the organic PD is operated under a reverse bias.^[^ ^8^ ^]^ The current−voltage (*I*_dark_−*V*) characteristics measured for the organic SWIR PDs with different BHJ layer thicknesses of 100, 150, and 200 nm in the absence of light are shown in **Figure** **3**a. Comparing to the PDs with a 100 nm thick BHJ layer, a clear decrease in the dark current in the organic SWIR PDs with a 200 nm thick BHJ is obtained, due to a noticeable reduction in the leakage current. PD with a low dark current is favorable for attaining a higher specific detectivity (D*). The noise current in the PDs includes the shot noise, thermal noise, 1/*f* noise, and generation‐recombination noise. The D* was calculated using the *R*(*λ*) characteristics and dark current measured for the organic SWIR PDs operated under different biases. The shot noise caused by dark current was considered as the main noise in the OPDs operated under the external bias.^[^ ^7^ ^]^ The shot‐noise‐limited D* is defined by: $D^{*}=R\left(\lambda \right)\sqrt{A}/{\left(2qI_{\mathrm{dark}}\right)}^{1/2}$, where *R*(*λ*) is the wavelength‐dependent photoresponsivity, *q* is the elementary charge, *A* is the active area, and *I*_dark_ is the dark current of the PD operated under a reverse bias. D* as a function of the wavelength, calculated for the organic SWIR PD with a 200 nm thick BHJ under different reverse biases, are plotted in Figure 3b. It shows that D* of the organic SWIR PDs is not very sensitive over the reverse bias range from −50 to −200 mV, with a D* of >10^9^ Jones over the long wavelength range from 1000 to 1400 nm, a very encouraging feature for practical application.

The reports on organic SWIR phototransistors (PTs) are relatively limited, due to the strict channel requirements, e.g., having both high carrier mobility and high SWIR light absorption. DPP‐DTT is a p‐type polymer semiconductor with a high carrier mobility,^[^ ^15^, ^16^, ^17^ ^]^ which has been adopted for use in organic NIR PTs.^[^ ^13^ ^]^ Taking advantage of the high carrier mobility of the DPP‐DTT polymer and the excellent absorption of the DPP‐DTT:SWIR dye blend layer over the long wavelength range from 1000 to 1600 nm, the suitability of the DPP‐DTT:SWIR dye BHJ for application in organic SWIR PTs has also been attempted. The cross‐sectional view of an organic SWIR PT, having a bottom gate top contact configuration, is shown in the inset in Figure S3a (Supporting Information). A 100 nm thick SWIR sensitive BHJ channel layer was overlaid on the surface of SiO_2_ gate dielectric. The heavily doped p‐type Si substrate serves as the gate electrode. The top gold (Au) source and drain contacts were used in the organic SWIR PTs. The photoresponsivity of the organic SWIR PTs was analyzed by monitoring the channel current (*I*_DS_) under different intensities of SWIR (1050 nm) light. Figure S3a (Supporting Information) plots the transfer characteristics of an organic SWIR PT measured in the dark and under SWIR (*λ* = 1050 nm) irradiation with different intensities of 0.11, 0.32, and 1.08 mW cm^−2^. A fixed source–drain bias (*V*_DS_) of −30 V was used in the measurements. In the presence of SWIR illumination, the dissociation of the excitons in the channel layer, generated due to the absorption of the SWIR (1050 nm) light in the active channel, contributes to an increase in the *I*_DS_ in the organic SWIR PT. The photocurrent is calculated by subtracting the *I*_DS_ obtained in the presence of SWIR illumination to the one measured in the absence of SWIR illumination. The photoresponsivity of the organic SWIR PTs as a function of the gate–source voltage (*V*_GS_) is shown in Figure S3b (Supporting Information). The organic SWIR PT had a higher photoresponsivity of 75 mA W^−1^, measured in the presence of a low SWIR intensity of 0.11 mW cm^−2^, operated under a *V*_GS_ of −30 V. It is clear that the photoresponsivity of the organic SWIR PT can be tuned from 16 to 75 mA W^−1^ when the intensity of SWIR illumination decreased from 1.08 to 0.11 mA cm^−2^, revealing its photodetection capability at low SWIR levels. The time response of the organic SWIR PT was analyzed using a 0.5 Hz modulated SWIR (1050 nm) source, the typical transient photoresponse profile of an organic SWIR PT is shown in Figure S3c (Supporting Information). The results reveal that the organic SWIR PT demonstrated in this work had an average rise time of ≈32 ms, as shown in Figure S3d (Supporting Information), which is >100 times faster than that of an organic NIR PT with a DPP‐DTT channel layer observed in our previous work.^[^ ^18^ ^]^

**Figure 4:**
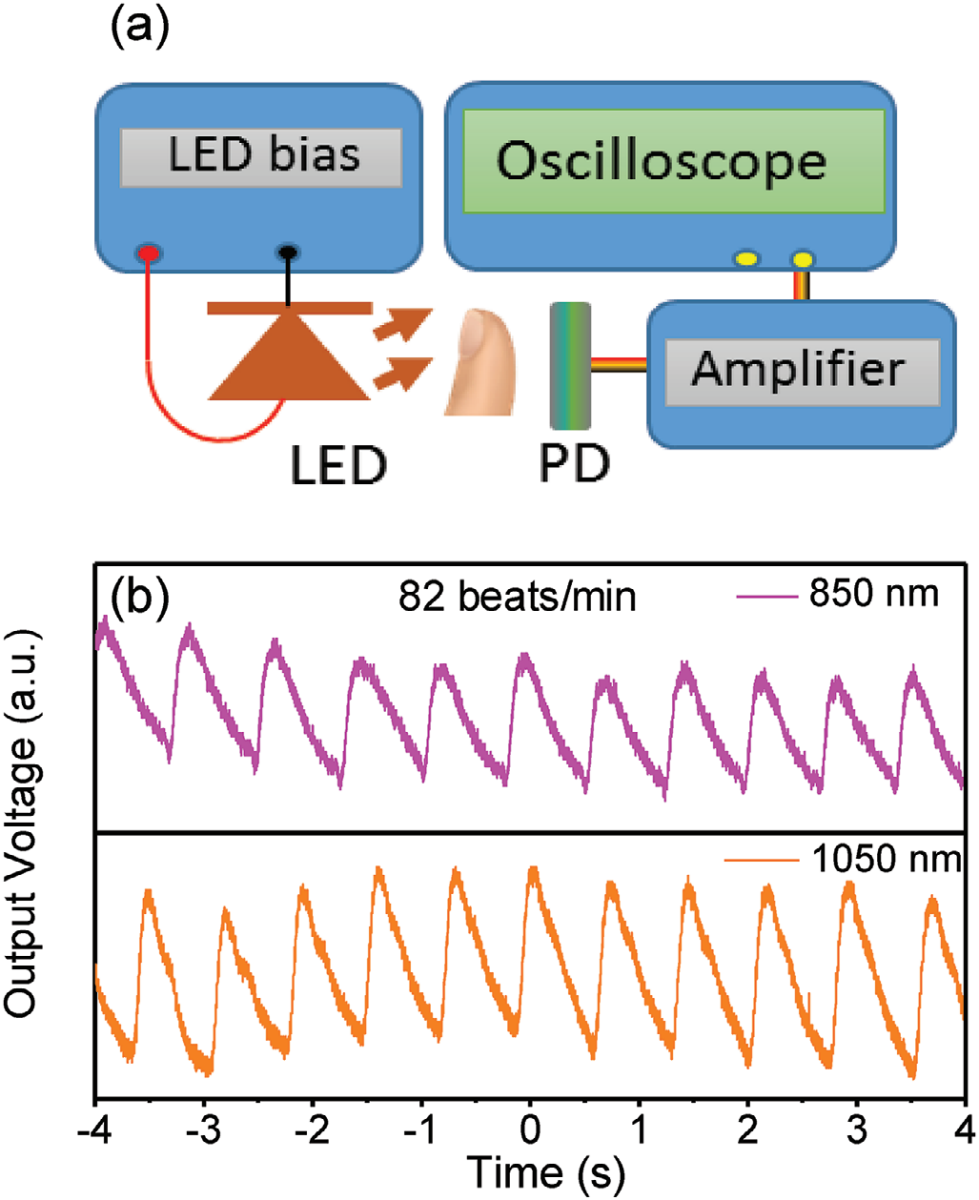
Organic SWIR PD‐based PPG sensor. a) A schematic PPG measurement setup comprising an infrared LED light source, an organic SWIR PD, an amplifier, and an oscilloscope. b) The heartbeat waveform measured by the organic SWIR PD‐based PPG sensor using NIR (850 nm) and SWIR (1050 nm) light illuminations, e.g., showing a heart rate of 82 beats min^−1^.

In comparison to the organic SWIR PTs, the organic SWIR PDs can be operated under a much lower bias, which is useful for portable and wearable photodetection and monitoring applications, e.g., the real‐time health monitoring and medical diagnose. The SWIR light is safe to use and has a deeper penetration depth in the biotissues compared to that of the NIR light. PPG sensor, the commonly used in pulse oximetry, measures noninvasively the variation in the arterial blood volume through monitoring the change in the intensity of the SWIR light that passes through the human tissue, e.g., fingertip or earlobe, monitoring real‐time heart rate and oxygen saturation. The transient photoresponse of an organic SWIR PD with a 200 nm thick BHJ layer was measured, validating its PPG sensor capability for measuring the heart rate. A schematic setup of the heart rate monitoring system through a fingertip is shown in **Figure** **4**a. In the PPG measurements, continuous NIR (850 nm) and SWIR (1050 nm) illuminations were used. The transient photoresponse characteristics of the organic SWIR PD were also studied using a modulated NIR (830 nm) LED light source with different frequencies of 100, 200, and 300 kHz, as shown in Figure S4 (Supporting Information), revealing that the organic SWIR PD had a fast photoresponse speed. The organic SWIR PD had a rise time and fall time of ≈1.0 µs, which is sufficient for application in heart rate monitoring where the operation frequency is of the order of 100 Hz. SWIR light has a deeper penetration depth in human tissues as compared to that of the NIR light.^[^ ^3^, ^4^ ^]^ The output signal from the organic SWIR PD was recorded using an oscilloscope. The output PPG signal measured using 850 and 1050 nm light sources is shown in Figure 4b. The effective PPG function in both the NIR and the SWIR wavelength ranges was clearly demonstrated. The use of SWIR light has the advantage in bioimaging and detection due to its deeper penetration depth in biotissues. The organic SWIR PD, with an encouraging photoresponse extending to 1600 nm, as demonstrated in this work, provides an opportunity for application in portable and wearable PPG sensory gadgets.

### 2.2 SWIR Visualization

The conventional infrared visualizing systems comprise an expensive III–V compound semiconductor infrared PD array and a signal‐processing unit. The images are displayed after the complicated data acquisition and processing. The inorganic semiconductor infrared PD array in these visualizing systems is fabricated primarily using an expensive and complex thin film epitaxial growth process. They are usually small in size, very fragile, and costly. Encouraging progresses have been made in the development of infrared‐to‐visible upconversion devices, e.g., coupling a colloidal lead selenide nanocrystal NIR harvesting layer with an OLED achieving a photon (1300 nm)‐to‐photon (520 nm) conversion efficiency of 1.3%,^[^ ^19^ ^]^ and combining a colloidal lead sulfide quantum dot (QD) infrared light sensitizing layer and a cadmium selenide/zinc selenide QD LED with a high photon‐to‐photon conversion efficiency of 6.5%.^[^ ^20^ ^]^ A high‐gain infrared‐to‐visible upconversion photodetector has also been demonstrated by incorporating a vertical infrared phototransistor with an OLED, attaining a high photon‐to‐photon conversion efficiency of more than 1000%.^[^ ^21^ ^]^

In addition to the advancements of the upconversion devices by combining inorganic infrared PDs, substantial research effort has also been devoted to improving the performance of NIR‐to‐visible upconversion devices through monolithic integration of organic NIR PDs and OLEDs or LEDs. Organic NIR PDs have attracted increasing interests due to the huge variety of material choices, versatile device fabrication capabilities, and large‐area processing flexibility. Recent progresses made in the NIR‐to‐visible upconversion devices through integrating different organic NIR PDs and OLEDs or LEDs are summarized in **Table** **1**. The SWIR‐to‐visible upconversion device with an organic SWIR PD demonstrated in this work has advanced the capability of detecting the longest infrared wavelengths up to 1400 nm as compared to that of the ones with an OPD reported in the field. The upconversion device with extension of the SWIR wavelengths (up to 1400 nm), which covers the spectrum of the second bioimaging window,^[^ ^29^ ^]^ can be very suitable for applications in medical and wellness monitoring, e.g., portable and wearable PPG sensory gadgets illustrated in Figure 4. SWIR visualizing devices demonstrated in this work were prepared using all solution fabrication process, which can be readily adopted for flexible large‐area SWIR‐to‐visible upconversion devices at a low cost.

**Table 1:** Summary of upconversion devices prepared through monolithic integration of different organic infrared PDs and LED components

Detection/emission wavelengths [nm]	OPD	Emission layer	Active area [cm × cm]	Upconversion efficiency [%]@V	Ref.
800 nm/green	ClAlPc:C_70_	CBP:Ir(ppy)_3_	0.04 × 0.16	6%@7 V	^[^ ^22^ ^]^
900 nm/green	PbPc:C_60_	Alq_3_	0.16 × 0.5	0.043%@28 V	^[^ ^23^ ^]^
900 nm/480, 512, 584 nm and white	ING‐T‐DPP: PC_60_BM	TADF materials	0.04 × 4.0	0.044–0.11%@10 V (W/W)	^[^ ^24^ ^]^
950 nm/green	PDPP3T:PC_61_BM	Be(pp)_2_:Ir(ppy)_2_(acac)	0.16 × 4.0	29.6%@12 V	^[^ ^25^ ^]^
1000 nm/green	SnPc:C_60_	Irppy_3_:CBP	0.04	2.7%@15 V	^[^ ^26^ ^]^
1000 nm/520 nm	DPP‐DTT: CO_i_8DFIC	CsPbBr_3_	0.3 × 0.3	1.9%@6 V	^[^ ^12^ ^]^
1050 nm/green	SnNcCl_2_:C_60_	CBP:Ir(ppy)_2_(acac)	0.02 × 1.44	5%@25.8 V	^[^ ^27^ ^]^
1100 nm/green	SQ‐880:PCBM	Alq_3_	1.6	0.27%@12 V	^[^ ^28^ ^]^
1400 nm/516 nm	DPP‐DTT:IR dye	CsPbBr_3_	1.0 × 1.5	0.1%@14 V	^This work^

The recent progresses made in the solution‐processable OPDs provide an encouraging pathway for application in large‐area SWIR visualization at a low cost. The monolithic integration of an organic SWIR PD and a visible light LED is capable of visualizing SWIR light through an upconversion process.^[^ ^12^, ^22^, ^26^ ^]^ Compared to the conventional III–V compound semiconductor PD array‐based infrared imaging systems, the most important attribute of the organic SWIR visualizing device is its large area and solution fabrication capability that can significantly reduce manufacturing cost.

**Figure 5:**
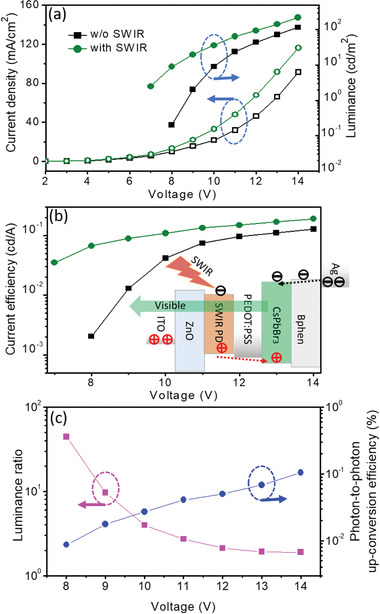
Properties of the SWIR visualizing device. a) *J*−*L*−*V* characteristics measured for the SWIR visualizing device in the absence and presence of SWIR (1050 nm) light with an intensity of 32 mW cm^−2^. b) *L*−*V* characteristics measured for the SWIR visualizing device in the absence and presence of the SWIR (1050 nm) light. Inset in (b): a schematic diagram illustrating the energy levels of the materials used in the SWIR visualizing device. c) The luminance ratio and photon‐to‐photon upconversion efficiency as a function of the operating voltage.

With the exciting progresses made in the solution‐processable organic SWIR PDs in this work, an all‐solution‐processable SWIR visualizing device has been demonstrated, realized by the monolithic integration of an organic SWIR PD and a perovskite LED. The organic SWIR PD acts as a charge generation layer in the SWIR‐to‐visible upconversion device. The CsPbBr_3_ perovskite LED with an emission area of 1.0 × 1.5 cm^2[^ ^30^ ^]^ was used in the SWIR visualizing device. The active area of the organic SWIR PD can be tailored easily according to different applications and is much larger than that of the inorganic SWIR PDs available in the market. The emission in the CsPbBr_3_ perovskite LED in the absence of the SWIR illumination is due to the leakage current formed in the organic SWIR PD in the upconversion device, operated under a high forward bias. The CsPbBr_3_ perovskite LED has an electroluminescence (EL) emission with a peak position at 516 nm, close to the peak sensitivity of the human vision, and an EL spectrum with a full width at half maximum of <24 nm. The EL spectra of the CsPbBr_3_ perovskite LED operated under different voltages are plotted in Figure S5a (Supporting Information). It displays a saturated green light with the CIE coordinates of (0.093, 0.734), as shown in Figure S5b (Supporting Information). The current density−luminance−voltage (*J−L−V*) characteristics measured for the SWIR visualizing device in the absence and presence of SWIR (1050 nm) light are shown in **Figure** **5**a. An obvious increase in the current density and luminance was observed for the SWIR visualizing device in the presence of the SWIR (1050 nm) light illumination.

The effect of the sensitivity of the organic SWIR PD on the luminous efficiency of the CsPbBr_3_ perovskite LED in the absence and presence of SWIR light was clearly manifested, as shown in Figure 5b. The luminous efficiency of the SWIR‐to‐visible upconversion device increased substantially upon SWIR (1050 nm) light illumination, particularly under a low operating bias. The charge injection in the CsPbBr_3_ perovskite LED in the SWIR‐to‐visible upconversion device is controlled by the photocurrent generated in the organic SWIR PD in the presence of the SWIR (1050 nm) light. The emission in the CsPbBr_3_ perovskite LED is observed in area where the effective charge injection takes place, controlled by the photocurrent generated by the organic SWIR PD in the presence of the SWIR light. The inset in Figure 5b illustrates schematically the modulation of the injection current in the SWIR visualizing device in the presence of SWIR light. The photon‐to‐photon upconversion efficiency and luminance ratio, defined by the ratio of the luminance measured for the SWIR visualizing device in the presence of SWIR light over the one measured in the absence of the SWIR light, as a function of the operation voltage are plotted in Figure 5c. The results reveal that the photon‐to‐photon upconversion efficiency in the SWIR visualizing device increases with the external bias, while the luminance ratio decreases with the external bias, due to the leakage current formed in the organic SWIR PD operated under a large forward bias. A photon‐to‐photon conversion efficiency of >0.1% is obtained for the SWIR visualizing device operated under 14 V. The ratio of SWIR photon to visible light photon upconversion in the SWIR visualizing devices is lower than that of the NIR photon to visible light photon upconversion in the NIR visualizing devices.^[^ ^12^, ^21^ ^]^ The photoresponse of a photodetector depends on the light absorption, exciton dissociation, and charge extraction efficiency. The lower SWIR to visible light upconversion efficiency is associated with the limited photoresponse in the organic SWIR PDs, due to the poor charge dissociation and charge collection efficiency. The effective internal electric field in the OPDs decreases with the decrease in the bandgap of the organic semiconductor, e.g., the use of a hetero‐polycyclic aromatic compound SWIR dye in this case, leading to a less efficient dissociation of charge transfer excitons, and thereby a small photocurrent.^[^ ^31^ ^]^ The low effective internal electric field also induces an undesired increase in charge recombination, resulting in a low photoresponse due to the poorer charge transport and charge extraction.^[^ ^32^, ^33^ ^]^

In parallel to the development of new molecules with excellent long wavelength sensitivity,^[^ ^14^ ^]^ the morphology and the vertical stratification of a BHJ and the configuration of the OPDs also play an important role in determining the performance of the upconversion devices, including charge transfer, recombination, and thereby the photodetection of the organic SWIR PDs.^[^ ^33^ ^]^ The photoresponse of the SWIR detection can also be increased by adopting a three‐terminal phototransistor device architecture, e.g., using a high‐gain infrared phototransistor for attaining high photon‐to‐photon conversion efficiency.^[^ ^21^ ^]^

**Figure 6:**
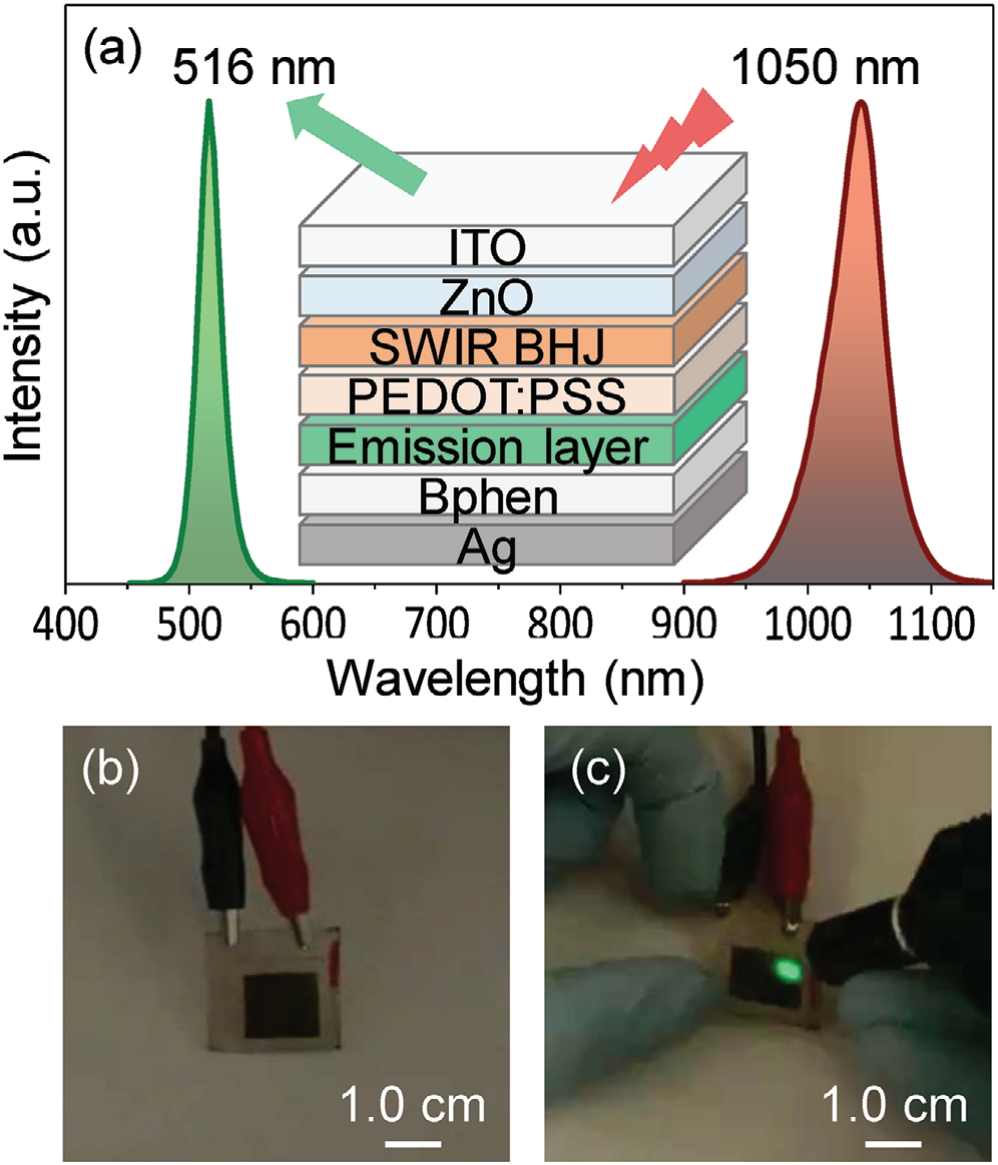
SWIR visualization. a) The spectrum of a SWIR LED source used in the measurement and the EL spectrum of the SWIR‐to‐visible upconversion device in the presence of the SWIR (1050 nm) light illumination, the inset in (a): a schematic cross‐sectional view of the SWIR visualizing device. b) The picture taken for the SWIR visualizing device, operated under a forward bias of 7.0 V, in the absence of SWIR illumination, showing no visible light emission. c) The picture taken for the SWIR visualizing device, operated under a forward bias of 7.0 V, in the presence of a circular SWIR (1050 nm) light source. The intensity of the SWIR (1050 nm) light was ≈35 mW cm^−2^.

The spectrum of a SWIR LED source used in the measurement and the EL spectrum of the visualizing device in the presence of the SWIR (1050 nm) light illumination are plotted in **Figure** **6**a, a schematic cross‐sectional view of the SWIR visualizing device is shown in the inset in Figure 6a. The picture in Figure 6b was taken for a SWIR visualizing device, operated under a forward bias of 7.0 V. No visible light emission was observed due to the lack of charge injection in the CsPbBr_3_ perovskite LED in the absence of SWIR illumination. The visible light emission in the SWIR visualizing device is realized only in area that is illuminated by the SWIR light, such that the objects reflecting or illuminating SWIR light can be visualized. To demonstrate the real‐time visualization of the invisible SWIR (1050 nm) light, a video taken for the SWIR‐to‐visible upconversion device, operated under a forward bias of 7.0 V, emitting the visible green light (516 nm) using a circular SWIR (1050 nm) light source is given in the Supporting Information. The corresponding picture taken for the SWIR device showing a visible emitting area that reflects the circular invisible SWIR (1050 nm) light source is shown in Figure 6c. The real‐time SWIR visualization was clearly demonstrated. This is the first report on visualization of SWIR light using an upconversion device fabricated by all‐solution‐based process.

The results of the SWIR visualization presented in this work are very encouraging, providing a platform technology for use in detection of SWIR absorbing materials embedded in the non‐SWIR absorbing materials, e.g., offering a fast detection approach for visualizing the SWIR‐absorbing microplastics^[^ ^34^ ^]^ that are ingested by the marine animals. The SWIR visualization device can also be used for detecting the leakage of the gases having SWIR‐absorbing characteristics.^[^ ^35^, ^36^ ^]^

## 3 Conclusions

In this work, we report our efforts to develop a high‐performance organic SWIR PD for photodetection and visualization. The organic SWIR PD has a photoresponsivity over a broadband wavelength range from 400 to 1600 nm, with an encouraging D* of >10^9^ Jones over the long wavelength range from 1000 to 1400 nm. The organic SWIR PDs thus developed are not sensitive in photoresponsivity over a working temperature range from 240 to 300 K and can be operated without cooling. An organic SWIR PD‐based PPG sensor is capable of providing a reliable heart rate monitoring using a SWIR (1050 nm) light source. A large‐area SWIR visualizing device, comprising a stack of an organic SWIR PD and a CsPbBr_3_ perovskite LED, has been demonstrated. The results presented in this work are very inspiring, providing a platform technology for application in large‐area SWIR photodetection and visualization at a low cost.

## Conflict of Interest

The authors declare no conflict of interest.

## Author Contributions

N.L. carried out most of the experiments (PD fabrication, PD/LED integration and characterization). Z.L. helped N.L. for the device measurement. Y.S.L. helped N.L. for the perovskite LED optimization and characterization. J.X. synthesized SWIR material under the supervision of D.Z. F.Z. directed the overall project and manuscript was written with contributions from all authors.

## Supporting information

Supporting Information

Supplemental Video 1
