# Supplementary material for: SWIR Photodetection and Visualization Realized by Incorporating an Organic SWIR Sensitive Bulk Heterojunction
Source: Adv Sci (Weinh). 2020 May 29;7(14):2000444. doi: 10.1002/advs.202000444 (PMC7375246; doi:10.1002/advs.202000444)
Supplement: Supplementary file 1 — Supporting Information [file ADVS-7-2000444-s001.pdf]

Supporting Information

SWIR Photodetection and Visualization Realized by Incorporating an Organic SWIR Sensitive Bulk Heterojunction

Ning Li[1], Zhaojue Lan[1], Ying Suet Lau[1], Jiajun Xie[2], Dahui Zhao[2]*, Furong Zhu[1]*

[1] Department of Physics, Research Centre of Excellence for Organic Electronics, Institute of Advanced Material, and State Key Laboratory of Environmental and Biological Analysis, Hong Kong Baptist University, Hong Kong, China

[2] Beijing National Laboratory for Molecular Sciences, Centre for Soft Matter Science and Engineering, Key Laboratory of Polymer Chemistry and Physics of the Ministry of Education, College of Chemistry, Peking University, Beijing, China.

* dhzhao@pku.edu.cn; frzhu@hkbu.edu.hk

1. Material preparation

The polymer (DPP-DTT, Ossila) and SWIR dye blend solution was prepared by dissolving the DPP-DTT:SWIR dye mixture (with a weight ratio of 1:1) in 1,2-Dichlorobenzene, having a concentration of 10 mg/mL. 1% (volume ratio) DIO (Sigma Aldrich) was then added in the blend solution. The blend solution was annealed at 50 ℃ and stirred overnight for improving the miscibility before use.

The $CsPbBr_3$ solution was prepared by dissolving 0.31 M CsBr (Sigma-Aldrich) and 0.18 M $PbBr_2$ (Sigma-Aldrich) in dimethyl sulfoxide (Sigma-Aldrich). Polyethylene oxide (PEO) additive was dissolved in dimethyl sulfoxide with a concentration of 20 mg/mL and stirred at 60 ℃ for 3 h. The $CsPbBr_3$ precursor solution was formulated by mixing $CsPbBr_3$ and PEO

solutions at a volume ratio of 10:1 and stirred for 1 h before use. The ZnO precursor solution was formulated with a concentration of 0.5 M in methoxyethanol (Sigma-Aldrich). All materials were used as received.

2. Device fabrication and characterization

Device fabrication: The pre-patterned ITO/glass substrates with a sheet resistance of 10 ohm/sq were cleaned by ultrasonication in detergent, deionized water, acetone and isopropanol each for 10 min. The ITO surface was modified with UV treatment for improving the hydrophilicity and removing the possible chemical residuals in the wet clean processes. A 50 nm thick ZnO was formed on the ITO surface using spin-coating. After annealing at 200 ℃ for 20 min, the substrates were transferred to glovebox with the O_2 and H_2O levels <0.1 ppm. The organic SWIR BHJ layer was formed on the ZnO surface. The thickness of the BHJ was controlled by adjusting the spin-coating speed. The BHJ layers were then annealed at 150 ℃ for 10 min. The samples were transferred to the vacuum chamber for depositing a 2.0 nm thick MoO_3 hole transporting layer and a 100 nm thick Ag electrode, forming the organic SWIR PDs. The PDs with two different active areas of ~0.1 and 1.5 cm^2 were prepared. The surface of the BHJ in the organic SWIR PDs was modified using argon plasma prior to the deposition of a 50 nm thick PEDOT:PSS layer by spin-coating. A 30 nm thick $CsPbBr_3$ emission layer was overlaid the surface of the PEDOT:PSS layer by spin-coating and was annealed at 70 ℃ for 5 min. The samples were then transferred to the vacuum chamber for deposition of a 100 nm thick Ag electrode by thermal evaporation. The active area of the SWIR-to-visible upconversion device is 1.5 cm^2.

The p-doped Si wafers with a 300 nm thick thermally grown SiO_2 were used for preparation of the organic SWIR PTs. The Si substrates were cleaned by ultrasonication sequentially in

deionized water, acetone and isopropanol, each for 10 min. The Si/SiO_2 surface was treated with UV to remove residuals. A 100 nm thick organic SWIR channel layer was formed on the Si/SiO_2 surface prepared by spin coating. Post-annealing of the active layer was performed at 150 ℃ for 10 min. The 60 nm thick Au source and drain contacts were prepared by thermal evaporation. The channel length in the organic SWIR PT was defined using shadow masks. The channel length and width of the organic SWIR PT were 80 and 1500 μm, respectively. Both organic SWIR PDs and SWIR-to-visible upconversion devices were encapsulated in the glovebox before taking out for the measurements. The organic SWIR PTs were measured in ambient without encapsulation.

PD characterization: The photoresponsivity of the organic SWIR PDs were measured using a xenon lamp light source with a monochrometer. The photocurrent of the organic SWIR PD was collected using a lock-in amplifier (Stanford Research Systems). The light intensity dependent *J*-*V* characteristics of the organic SWIR PDs were measured using different LED (Zolix Instruments CO., LTD) light sources with peak wavelengths at 365, 450, 850 and 1050 nm. The photocurrent of the organic SWIR PDs was recorded by an electrometer (Keithley 2636B). The temperature dependent photoresponse characteristics of the organic SWIR PDs were measured using a cryostat (Oxford).

PT characterization: The V_{GS} and V_{DS} were supplied by an electrometer (Keithley 2636B). The channel current I_{DS} was measured and recorded by the same electrometer. A SWIR (1050 nm) LED light source (Zolix Instruments CO., LTD) was used, the light intensity of the SWIR LED source was varied using optical attenuators.

PPG measurements: The PPG measurements were performed by measuring the light signal transmitted through the fingertip using the organic SWIR PD operated without an external bias. The optical signal was provided by LED sources with different peak wavelengths of 850 and 1050 nm. The photocurrent of the organic SWIR PD was amplified by a low noise preamplifier (Stanford Research Systems). The PPG signals were displayed and recorded using an oscilloscope (Tektronix MDO 3052).

SWIR visualization: $J-L-V$ characteristics of the SWIR-to-visible upconversion devices were measured using a luminance colorimeter, an electrometer and recorded using the operating software. The SWIR visualizing device was demonstrated using circular SWIR (1050 nm) light source.

3. Supporting figures:

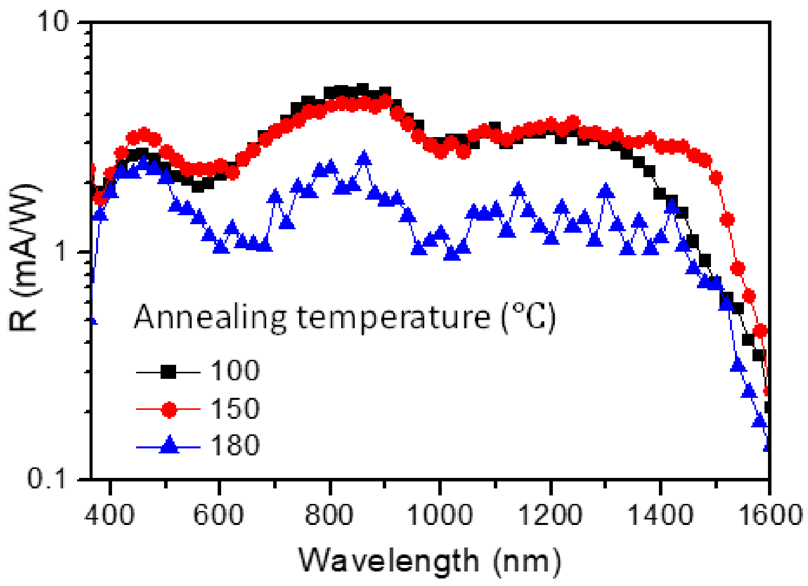

Figure S1. The photoresponsivity measured for the organic SWIR PD with a 200 nm thick BHJ layer, annealed at different temperatures of 100, 150 and 180 °C.

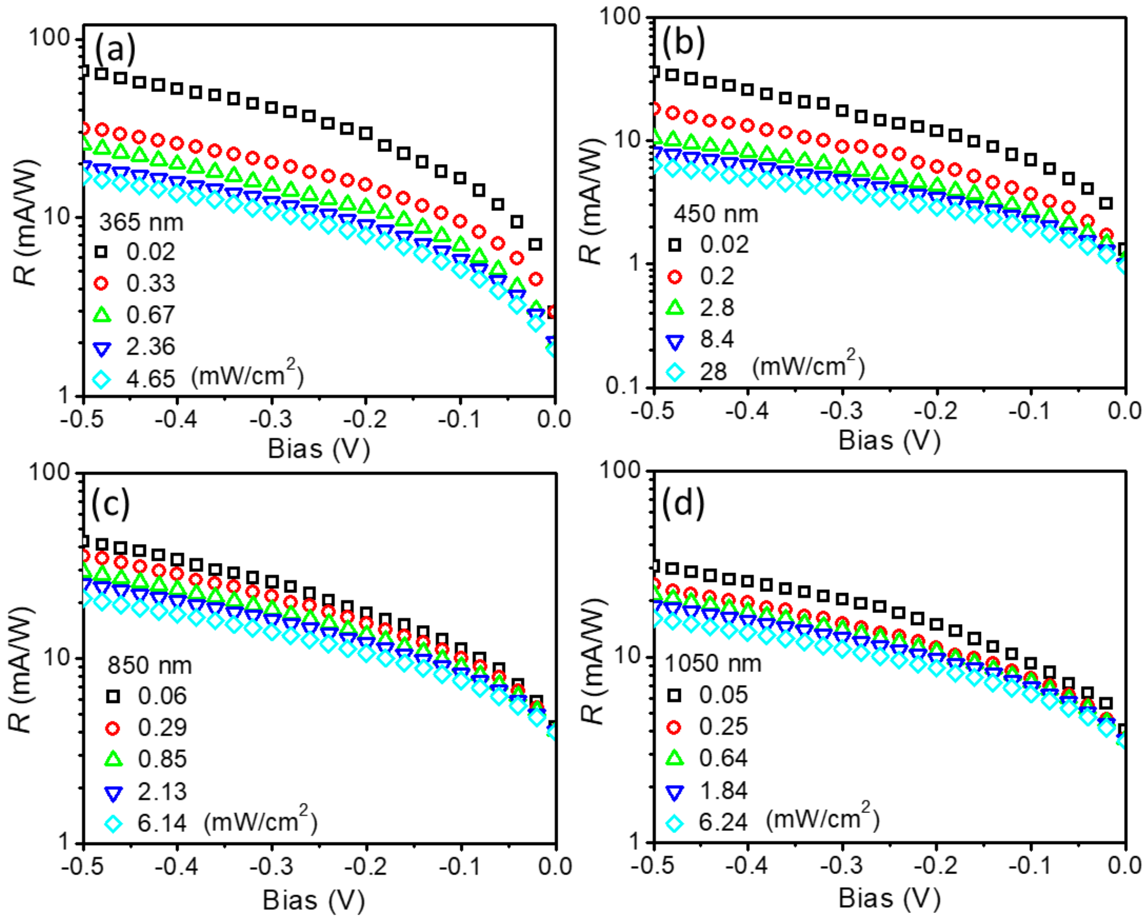

Figure S2. The photoresponsivity of the organic SWIR PD with a 200 nm thick active layer, measured under different intensities of the light sources with different peak emission wavelengths of (a) 365, (b) 450, (c) 850 and (d) 1050 nm.

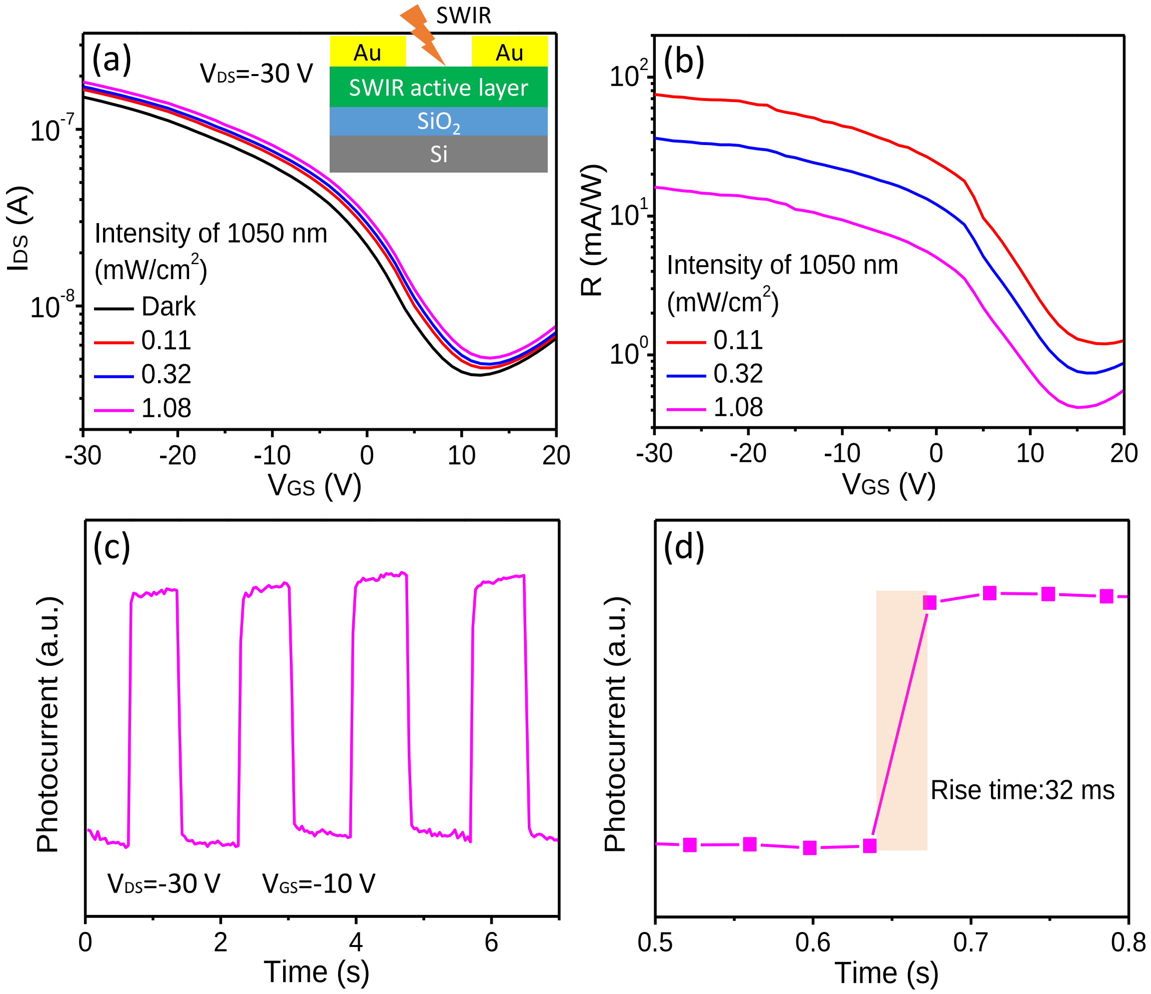

Figure S3. (a) I_{DS}–V_{GS} characteristics measured for the organic SWIR PTs under different intensities of the SWIR (1050 nm) light. V_{DS} was fixed at -30 V. The inset in (a): a schematic cross-sectional view of the organic SWIR PT. (b) $R(\lambda)$ of the organic SWIR PTs as a function of the intensity of the SWIR (1050 nm) light. (c) The transient photoresponse measured for the organic SWIR PT using the modulated SWIR (1050 nm) light. (d) Transient photocurrent response curve measured for the organic SWIR PT, estimating a response time of 32 ms.

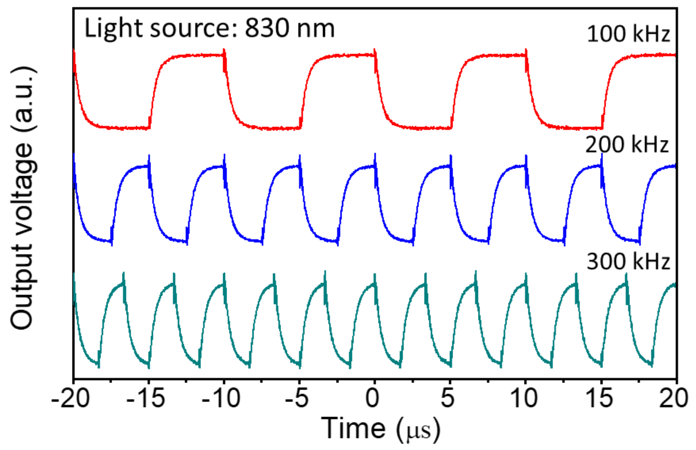

Figure S4. The transient photoresponse characteristics measured for the organic SWIR PDs in the presence of NIR light (830 nm) modulated at different frequencies of 100, 200 and 300 kHz, with a rise time and a fall time of ~1.0 μs.

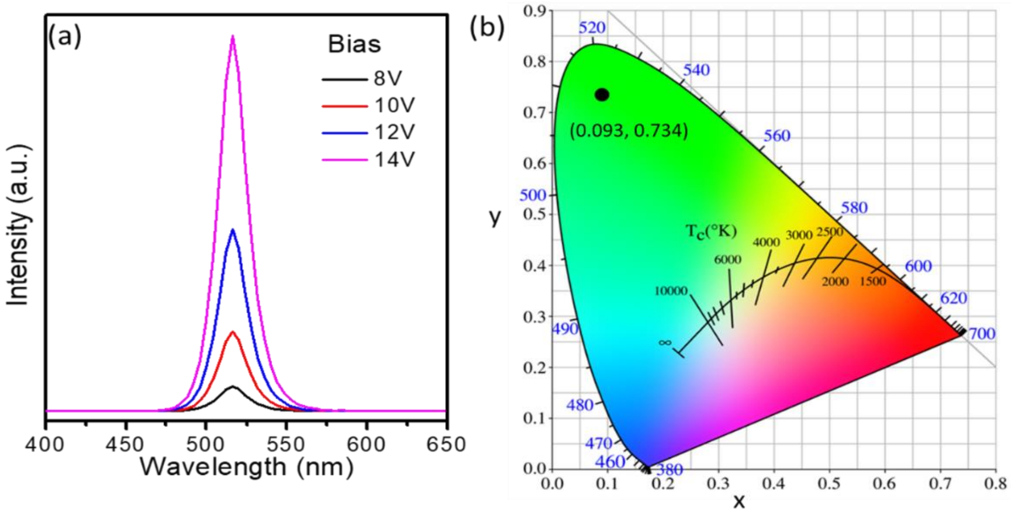

Figure S5. (a) EL spectra measured for the SWIR visualizing device at different forward biases of 8.0, 10.0, 12.0 and 14.0 V in the presence of SWIR (1050 nm) light. (b) The CIE coordinates of EL spectrum measured for the SWIR visualizing device.

Video: a video taken for the SWIR-to-visible upconversion device, operated under 7.0 V, using a circular SWIR (1050 nm) light source, demonstrating visualization of SWIR light.
